# Prosexual Effect of* Chrysactinia mexicana* A. Gray (Asteraceae), False Damiana, in a Model of Male Sexual Behavior

**DOI:** 10.1155/2016/2987917

**Published:** 2016-08-30

**Authors:** R. Estrada-Reyes, O. A. Ferreyra-Cruz, G. Jiménez-Rubio, O. T. Hernández-Hernández, L. Martínez-Mota

**Affiliations:** ^1^Laboratorio de Fitofarmacología, Dirección de Investigaciones en Neurociencias, Instituto Nacional de Psiquiatría Ramón de la Fuente Muñiz, Calzada México-Xochimilco 101, Col. San Lorenzo Huipulco, Delegación Tlalpan, 14370 Ciudad de México, Mexico; ^2^Laboratorio de Farmacología Conductual, Dirección de Investigaciones en Neurociencias, Instituto Nacional de Psiquiatría Ramón de la Fuente Muñiz, Calzada México-Xochimilco 101, Col. San Lorenzo Huipulco, Delegación Tlalpan, 14370 Ciudad de México, Mexico; ^3^CONACYT Research Fellow, Instituto Nacional de Psiquiatría, Ciudad de México, Mexico

## Abstract

*Chrysactinia mexicana* A. Gray (Asteraceae) and* Turnera diffusa* Willd (Turneraceae) are employed in traditional medicine as aphrodisiacs; however, there is no scientific evidence supporting the prosexual properties of *C. mexicana*. The aim of this study was to determine whether an aqueous extract of* C. mexicana* (Cm) stimulates rat male sexual behavior in the sexual exhaustion paradigm. Sexually exhausted (SExh) male rats were treated with Cm (80, 160, and 320 mg/kg), an aqueous extract of* T. diffusa *(Td), or yohimbine. The sexual exhaustion state in the control group was characterized by a low percentage of males exhibiting mounts, intromissions, and ejaculations and no males demonstrating mating behavior after ejaculation. Cm (320 mg/kg), Td, or yohimbine significantly increased the proportion of SExh rats that ejaculated and resumed copulation after ejaculation. In males that exhibited reversal of sexual exhaustion, Cm (320 mg/kg) improved sexual performance by reducing the number of intromissions and shrinking ejaculation latency. The effects of treatments on sexual behavior were not related with alterations in general locomotion. In conclusion, the prosexual effects of Cm, as well as those of Td, are established at a central level, which supports the traditional use of* C. mexicana* for stimulating sexual activity.

## 1. Introduction 

Sexual interaction is an important factor for social and biological relationships in human life. Alterations in sexual activity may affect not only the health, but also the quality of human life. Studies carried out in the U.S. have estimated that up to 52% of men between ages 40 and 70 years of age suffer from some alterations of sexual health due to aging or resulting from medical conditions such as diabetes, stress, hypertension, or obesity [1]. In this case, medicinal plants comprise an alternative for improving sexual health, facilitating aspects of sexual performance, and increasing the libido [2]. This information derives from ethnomedical reports, which attribute prosexual effects to a group of plants, although scientific information of their pharmacological properties is not widely published.

Within the context of traditional medicine, the term “aphrodisiac” is given to any substance (i.e., food, beverages, or drugs) capable of stimulating the libido or sexual activity [3]. Investigation suggests that these substances may act at the level of the Central Nervous System (CNS) by altering specific neurotransmitters or sex hormones [3]. In this respect, herbal preparations considered as aphrodisiacs in traditional medicine may produce their effects by stimulating targets in the CNS.* C*.* mexicana* is a plant used in Mexican traditional medicine to treat a number of diseases, such as respiratory diseases, skin infections, and rheumatism, and as a diuretic and spasmolytic agent [4]. However, it is primarily used as a stimulating agent and as a “powerful aphrodisiac” to improve libido in males [5, 6]. In North America,* C. mexicana* is widely distributed from New Mexico and Texas south to central Mexico, including Chihuahua, Nuevo León, Durango, Zacatecas, Aguascalientes, San Luis Potosí, Guanajuato, Mexico City (CDMX), Hidalgo, Puebla, Veracruz, and Oaxaca. Due to its putative aphrodisiac properties,* C. mexicana* is highly consumed and commercialized in North America and some countries of South America.

*C. mexicana* is known as Damiana, Damianita, or false Damiana due to its morphological resemblance to “Damiana de California” (*Turnera diffusa* Willd, Turneraceae) and “*garañona*”, in reference to its proposed prosexual effects [5, 7]. Both species share the yellow color of their flowers and fragrance, but they possess morphological differences, that is, shape of leaves and stems. In both species, there is also chemical similarity, that is, apigenin, and its glucoside derivatives are present in each in significant amounts. However, unlike* C. mexicana*,* T. diffusa* has been identified as a plant with properties that stimulate sexual activity in different experimental models. Crude extracts of* T. diffusa* have been proven to stimulate sexual behavior in male rats classified as sexually sluggish, that is, those with slower copulation that requires more intromissions to reach the ejaculatory threshold. In these animals, extracts of* T. diffusa* reduced ejaculatory latency [8, 9]. Besides, in a previous study, we showed that a standardized aqueous extract of* T. diffusa* improved sexual behavior in sexually exhausted (SExh) male rats, reversing the inhibition of sexual behavior presented by these animals [10]. Sexual exhaustion (also denominated sexual satiation) is considered a paradigm of the central inhibition of male sexual behavior [11], which led us to propose that the extract of* T. diffusa* stimulates copulation by stimulating brain targets that participate in male sexual behavior.

Sexual exhaustion in male rats is a phenomenon that appears after sustained copulation with a single sexually receptive female. Sexual satiation has been mainly studied in male rats, and males of other mammalian species, such as hamster [12], guinea pig [13], rabbit [14], and Rhesus macaque [15] demonstrated similar behavior after sustained sexual activity. The standard protocol of sexual exhaustion in rats consists of free copulation during a 4 h period during which male rats achieve a mean of seven ejaculations [16]. There is evidence that copulation to satiation induces physiological changes that include transient modifications in brain functions [17, 18]. Thus, 24 h after* ad libitum* mating behavior, males present an inhibition of sexual behavior that can last up to 96 h [11, 19]. Several pharmacological studies have shown that sexual exhaustion is reversed by drugs that affect different neurotransmiter systems, such as the serotonergic (i.e., with the 5-HT_1A_ agonist, 8-OH-DPAT), noradrenergic (i.e., with the *α*2-adrenergic antagonist, yohimbine), and the glutamatergic (i.e., with antagonists of the NMDA, AMPA, and mGluR5 receptor subtypes) systems, which restore the expression of copulatory behavior [16, 23, 24, 27, 29]. Regarding the effects of* T. diffusa*, our previous findings support the fact that the sexual exhaustion paradigm is a good predictive model for the study of plants with putative aphrodisiac properties [10].

The aim of this research was to evaluate the prosexual effects of a standardized aqueous extract of* C. mexicana* (Cm) in SExh male rats. The actions of this extract were compared with those produced by a standardized aqueous extract of* T. diffusa* (Td) and yohimbine, employed as a reference drug for the reversal of sexual satiation [20]. Additionally, the effects of treatments in the Open Field Test (OFT) were determined for discarding nonspecific effects (i.e., changes in locomotor and general activity) that could affect the interpretation of results in the sexual behavior test.

## 2. Materials and Methods

### 2.1. Animals

Adult male (*n* = 69; 300 g body weight (BW)) and female (*n* = 40; 200 g BW) Wistar rats (obtained from the Vivarium of the Instituto Nacional de Psiquiatría “Ramón de la Fuente Muñiz” in Mexico City) were used in this study. Rats were housed (five animals per cage according to sex, 53 × 43 × 20 cm) in independent rooms with an inverted 12-h : 12-h light/dark cycle (lights on 22:00 h) and controlled conditions of temperature and humidity. Rats had free access to water and food during all experiments. Animal management was conducted according to the specifications of Mexican Official Norm (NOM-062-ZOO-1999) and the General Principles of Laboratory Animal Care (U.S. National Institutes of Health (NIH) publication #85-23, revised in 1985). The protocol was approved by the local Ethics Committee.

### 2.2. Vegetal Material and Preparation of the Aqueous Extract

*Chrysactinia mexicana* A. Gray (Asteraceae) aerial parts were collected in State of Morelos, Mexico. The aerial parts of* Turnera diffusa* Willd (Turneraceae) were collected in the State of Hidalgo, Mexico (voucher nos. 14407 and 11486, resp.). The aqueous extracts evaluated here were obtained and chemically characterized previously by our work group [10, 21, 22]. Briefly, aerial parts of either species were air-dried and finely ground to prepare the aqueous extract with 10 g of vegetal material per 90 mL of boiling, distilled water and heated for 10 min. The extract was allowed to cool at room temperature, filtered, and dried in a Telstar Freezer Dryer at −50°C and 0.01 mBar. The yield was 17.6% for Cm and 20% for Td of dried aqueous extracts, which were stored at −4°C.

### 2.3. Treatments and Experimental Design

Aqueous extracts (Cm and Td) were dissolved in saline 0.9% solution with one drop of Tween 80; this later solution was used as vehicle (Sigma-Aldrich, St. Louis, MO, USA), while yohimbine (Sigma-Aldrich) was dissolved in saline solution.

Independent groups of SExh males were* per os* (p.o.) treated with the Cm extract at 80 (*n* = 7), 160 (*n* = 10), and 320 (*n* = 11) mg/kg or the vehicle (*n* = 20), 60 min prior to starting the test. Doses were chosen in pilot studies carried out at our laboratory. Other groups of SExh males were treated with Td (80 mg/kg; p.o., *n* = 11; 60 min before the test) or yohimbine (2 mg/kg; i.p., *n* = 10; 30 min before the test); these doses have been reported to reverse the sexual inhibition of sexually satiated males [10, 23].

### 2.4. Sexual Behavior Evaluation 

#### 2.4.1. Training of Sexual Behavior

Male rats were trained for sexual experience in five sessions, one session per week, with sexually receptive females. For induction of receptivity, ovariectomized female rats were treated in a sequential manner with estradiol benzoate (Sigma-Aldrich, St. Louis, MO, USA; 4 *μ*g/rat, subcutaneously (s.c.)) and progesterone (Sigma-Aldrich; 2 mg/rat, s.c.) 48 and 4 h, respectively, prior to each evaluation of sexual behavior.

Training sessions were carried out in polycarbonate cylindrical cages (60 cm × 40 cm), under dim red light conditions, 3 h after the beginning of the photoperiod dark phase. Males were allowed to copulate until they achieved ejaculation in a 30 min session. Males that accomplished sexual behavior achieving ejaculation in a period of <15 min in at least three training sessions were considered sexually experienced and were subjected to sexual exhaustion.

#### 2.4.2. Sexual Exhaustion Paradigm

One week after the last sexual behavior training, sexually experienced males were allowed to freely copulate during a 4 h period with a single sexually receptive female. According to previous reports using this procedure [16], the sexual exhaustion state was confirmed by a period of 90 min without expression of male sexual behavior.

#### 2.4.3. Sexual Behavior Test

Twenty four h after copulation* ad libitum*, males were treated and evaluated in a standard sexual behavior test with a maximal length of 30 min. A novel sexually receptive female was used to stimulate sexual behavior in males. The sexual behavior test was immediately finished after the following: (a) the occurrence of the first copulatory series in the 30 min test: a copulatory series includes the ejaculatory series (from the first mount or intromission to ejaculation) plus the subsequent first intromission; (b) if ejaculation latency exceeded 30 min; or (c) if males did not express sexual behavior within 20 min. Two observers quantified three components of male sexual behavior: (a) mounts, defined as pelvic thrusting without intravaginal penile insertion; (b) intromissions, defined as pelvic thrusting with intravaginal penile insertion, and (c) ejaculation, pelvic thrusting with intravaginal expulsion of seminal contents. These observations were employed to calculate the percentage of animals that expressed mounts, intromissions, ejaculations, and resumption of copulation, as well as the total number of mounts (NM) and intromissions (NI) that preceded ejaculation; intromission latency (time from introduction of female to experimental cage to the occurrence of the first intromission, IL), ejaculation latency (time from the first intromission to male achieving ejaculation, EL), and postejaculatory interval (time from ejaculation to first intromission of a second ejaculatory series, PEI). Results were expressed as mean ± standard error of the mean (SEM).

The effect of treatments on male sexual behavior within the context of sexual satiation was interpreted according to Rodríguez-Manzo [24]. Facilitation of the expression of male sexual behavior was reported when treatments increased the proportion of males able to execute mounts and intromissions and to achieve ejaculation. Reversal of sexual exhaustion was determined by a significant increase in the percentage of rats able to resume copulation after ejaculation (from the beginning of copulation taking the first intromission into account). Additionally, improvement of sexual performance was determined by a reduction in NM and NI and a shortening of IL, EL, and PEI.

### 2.5. Open Field Test (OFT)

In order to discard possible unspecific effects due to drugs, actions of treatments on ambulation were evaluated in an OFT. One week after the final sexual behavior evaluation, male rats were randomly assigned to the treatments (*n* = 6 rats per group) following a Latin square design, in which each rat has the same opportunity to receive any treatment. One h after drug administration, the rats were tested in a Plexiglass cage with the floor divided into 12 equal squares. The number of squares that animals crossed during a 5 min period was recorded by two trained observers. Results were expressed as mean number of counts ± SEM.

### 2.6. Statistics

The proportion of copulating animals was analyzed utilizing the Fisher* F*-test. Data that meet the criteria of linearity and equality of variance were analyzed using parametric tests, while data that did not meet them were analyzed with nonparametric tests. Effect of Cm on the specific parameters of sexual behavior, that is, EL, was analyzed with a Kruskal-Wallis test followed by Dunn's test. Paired comparisons between each dose of Cm or vehicle versus Td or yohimbine were carried out with a Mann Whitney* U* test. Effect of Cm on ambulatory activity was analyzed with a one-way ANOVA followed by the correction of Bonferroni. Paired comparisons between each dose of Cm or vehicle versus Td or yohimbine were carried out with a Mann Whitney* U* test.

## 3. Results 

Twenty four h after prolonged copulation, sexually satiated males of the control group (rats treated with the vehicle) showed an inhibition of sexual behavior determined by a reduction in the number of subjects able to demonstrate regular sexual behavior. We found that <45% of the control group population exhibited mounts and intromissions, while only 35% of this group was able to achieve one ejaculation (Table 1). None of these SExh males was able to restart copulation after the single ejaculation.

**Figure 1:**
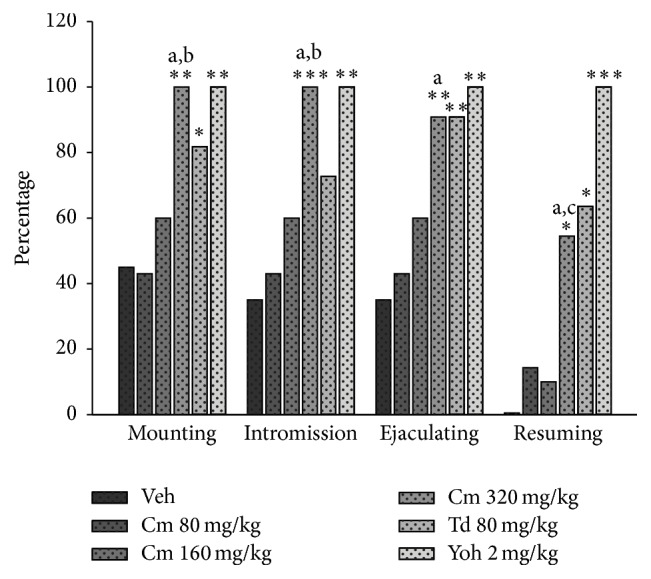
Effect of an aqueous extract of* C. mexicana* (Cm),* T. diffusa* (Td), or yohimbine (Yoh) on the percentage of sexually exhausted males that display sexual behavior. Results of the Fisher* F*-test: ^*∗*^ *p* < 0.05; ^*∗∗*^ *p* < 0.01; ^*∗∗∗*^ *p* < 0.001 versus the vehicle (Veh) control group; (a) *p* < 0.05 versus Cm 80 mg/kg group; (b) *p* < 0.05 versus Cm 160 mg/kg group; (c) *p* < 0.01 versus Cm 160 mg/kg.

Treatments utilized here stimulated SExh males to engage in one or more behaviors within the context of copulation. Male rats treated with Cm or Td extracts or yohimbine exhibited a normal pattern of movements during the course of copulation, which was not different from that shown by satiated males receiving the vehicle, or even from sexually experienced males (observations from the same males prior to the sexual exhaustion test). Thus, treated rats did not exhibit hyper- or hypoactivity nor stereotyped or circling movements in the sexual behavior test as compared with those treated with vehicle. In this evaluation, it was observed that the medium dose of Cm (160 mg/kg) produced a slight increase in the number of SExh subjects that could copulate until reaching ejaculation, although this effect did not attain statistical significance with respect to the vehicle control group (Figure 1). Clearly, the highest dose of Cm (320 mg/kg) significantly increased the proportion of rats that exhibited mounts, intromissions, and ejaculation. This latter dose also increased the proportion of males that restarted copulation after ejaculation. The effect of Cm on the expression of sexual behavior was doses-dependent in the percentage of animals that executed mounts and intromissions.

Regarding comparison among drugs, we found that the proportion of sexually satiated males that intromitted and ejaculated was similar under the treatment with Cm (320 mg/kg), Td, and yohimbine. In animals demonstrating mounts, such equivalence was found in the groups treated with Cm (320 mg/kg) and yohimbine. Regarding the percentage of animals that resumed sexual behavior, Cm (320 mg/kg) and Td produced similar effects. As expected, yohimbine produced the maximal effect on male sexual behavior, since 100% of treated animals showed mounts and intromissions, reached ejaculation, and resumed copulation.

**Figure 2:**
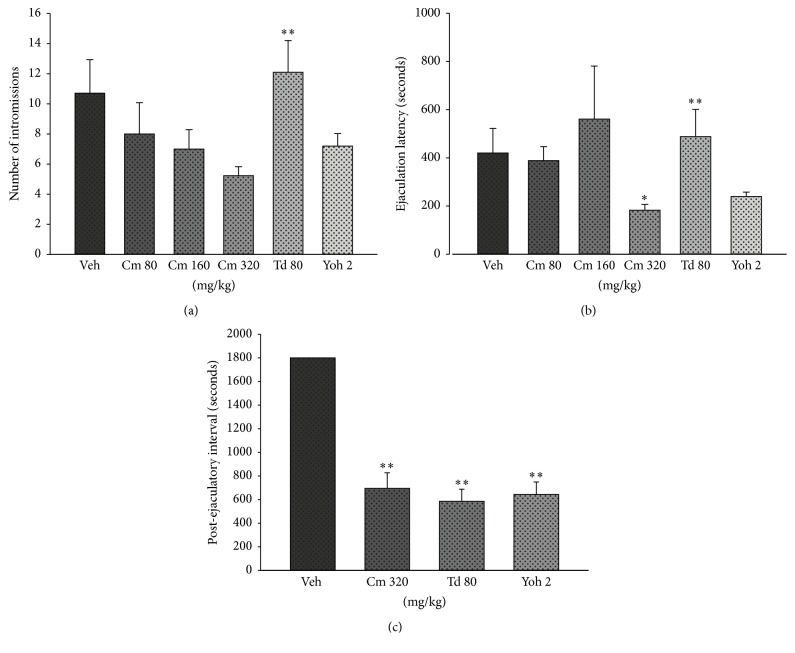
Effect of an aqueous extract of* C. mexicana* (Cm),* T. diffusa* (Td), or yohimbine (Yoh) on the number of intromissions (a), ejaculation latency (b), and postejaculatory interval (c) of sexually exhausted males that showed sexual behavior. Data of IPE were compared against an arbitrary value corresponding to the total time for a sexual behavior test. Results are expressed as mean ± S.E.M. Paired comparisons in groups treated with Cm were done with Dunn's test. Other comparisons were done with the Mann Whitney* U* test: in (a) ^*∗*^ *p* < 0.05 versus vehicle (Veh), ^*∗∗*^ *p* < 0.01 versus Cm 320 mg/kg; in (b) ^*∗*^ *p* < 0.05 versus Veh, ^*∗∗*^ *p* < 0.01 versus Cm 320 mg/kg; in (c) ^*∗∗*^ *p* < 0.01 versus Veh.

Analysis of the specific parameters of male sexual behavior was performed only in SExh rats that exhibited sexual behavior. Treatment with Cm produced no significant effects on IL (*H * = 0.18, df 3, *p* = 0.98), NM (*H * = 1.10, df 3, *p* = 0.77) (Table 2), and NI (*H * = 5.90, df 3, *p* = 0.11) (Figure 2(a)). However, paired comparisons showed that Td increased the NI with respect to the highest dose of Cm (320 mg/kg). Td did not increase the NI with respect to the vehicle control group (Figure 2(a)). Treatment with Cm significantly reduced the EL (*H* = 8.64, df 3, *p* = 0.034; Figure 2(b)) of SExh males, reaching the statistical significance with that higher dose of Cm (320 mg/kg) with respect to the vehicle control group.

In the vehicle-control group, no satiated male was able to normally copulate with fresh, sexually receptive females. This means that males exhibited a sexual exhaustion state and that their PEI could be as long as 72 h [11]. Thus, the effects of the treatments on PEI (Figure 2(c)) were compared versus an arbitrary value corresponding to the total evaluation time in the standard sexual behavior test (1,800 sec). Under these conditions, paired comparisons showed that Cm at higher dose, Td, and yohimbine significantly reduced the PEI regarding this arbitrary value. Medium and lower doses of Cm were not included in the analysis, because only one subject per group was able to resume copulation after ejaculation.

Treatment with Cm produced significant changes (*F * = 5.70, df 3, *p* = 0.005, Table 3) on the ambulatory activity evaluated in the OFT. Lower dose (80 mg/kg) of Cm increased the ambulatory activity and this effect was statistically different with respect to the same extract at 160 mg/kg or with respect to Td. Effect of Cm at lower dose was not significantly different from the vehicle control group. In this test treatments did not induce alterations in general motor activity, that is, stereotypes or circling behavior.

## 4. Discussion

Many herbal therapies show some potential benefits in improving male sexual function [25]; however, the therapeutic potential of these medicinal plants requires the support of the scientific evidence for validating their putative effects and safety. Our results represent the first evidence that an aqueous extract of* C. mexicana* is able to improve sexual behavior expression in male rats exhibiting transient inhibition of sexual activity.

Animal models to evaluate male sexual behavior in the laboratory usually constituted of rodents expressing different levels of sexual activity, such as sexually experienced or sluggish rats, which have shorter or longer ejaculation latencies, among other features [26]. On the other hand, the main feature of the sexual exhaustion paradigm is the central inhibition of male sexual behavior stimulated by repeated copulation with a single sexually receptive female [16, 23, 27]. According to the literature, sexual satiation involves three phases [28]. During the first phase, the majority of male rats (>92%) copulating* ad libitum* display a minimum of six ejaculations before reaching sexual satiety. This phase is followed by the phase involving the installation of sexual satiation, which requires a period of 24 h, during which brain plastic changes take place. The third phase is characterized by inhibition of sexual behavior during which only 30% of the SExh population can achieve a single ejaculation without restarting sexual activity after this. Results of the present study are in agreement with this information, in that the sexually competent males utilized for our experiment presented all phases until reaching the sexual exhaustion state by means of free copulation. Once satiety was installed, only 35% of the SExh population was able to achieve a single ejaculation, while no rat resumed copulation after ejaculation. The literature reports that this inhibition is well established during 24 h period after* ad libitum* mating behavior [11], whereby we used this period to evaluate the possible prosexual effects of Cm associated with its interaction with central targets in the nervous system.

Current experiments revealed that in SExh males the treatment with an aqueous extract of* C. mexicana* produces stimulant effects on sexual behavior. It was found that the medium dose of the extract (160 mg/kg) slightly increased the number of rats that showed mounts and intromissions and that attained threshold for ejaculating once. These effects attained the statistical significance with Cm at 320 mg/kg; therefore, this aqueous extract, at the highest dose employed herein, favored the expression of the male sexual behavior of SExh animals. Furthermore, this dose of Cm also increased by 54% the proportion of males that resumed copulation with respect to the control group (0%). According to the interpretation of sexual exhaustion paradigm and its regulation by drugs, a treatment is considered to reverse sexual satiety if it significantly increases the proportion of males that achieve ejaculation and resume copulation after ejaculation [16]. This pharmacological effect is produced by an ample number of drugs that affect different neurotransmitter systems [11, 29, 30]. In agreement with these, the present results provide evidence that the standardized extract of Cm at a high dose produces the reversal of sexual exhaustion state.

In traditional medicine,* C. mexicana* and* T. diffusa* are used interchangeably for improving sexual activity based on their botanical resemblance [7, 31]. Therefore, in the present study, the prosexual effects of Cm were compared with those of Td and yohimbine, employed as a reference drug with known effects on sexual exhaustion [20]. Our results demonstrated that both extracts improved the expression of sexual behavior in an equivalent manner, that is, allowing 90.9% of the population of satiated males to achieve ejaculation. The extracts also produced similar effects on the reversal of sexual satiation, because Cm increased the percentage of SExh males that resumed sexual activity at 54.5%, while Td did so at 63.6%. These results showed that the effective dose of Cm for reversal of sexual exhaustion was three-times higher (320 mg/kg) than that of Td (80 mg/kg). This difference in pharmacological potency may have implications for the ethnomedical use of species as aphrodisiac remedies. As expected, yohimbine at 2 mg/kg was the most effective drug for promoting the resumption of copulation in SExh rats. This result agrees with data from previous literature demonstrating the high efficacy of yohimbine in the reversal of sexual satiation [16]. Differences in the effective treatment doses utilized here for the reversal of sexual satiation could be explained taking into account that yohimbine is a pure drug, while crude extracts are constituted of multiple components [10, 21] of which their pharmacokinetic and pharmacodynamics are unknown.

In relation to the sexual performance of SExh rats that recovered sexual activity, only Cm at the highest dose (320 mg/kg) produced further effects on male sexual behavior, reducing NI and shortening EL. Consistently, Cm (320 mg/kg), Td, and yohimbine produced a reduction of the PEI of sexually exhausted males, in comparison with an arbitrary value corresponding to total time of test. This value was established considering that satiated control-group were unable to resume copulation during the test and that the reestablishment of normal sexual activity could take >72 h after free copulation [11]. Then, in addition to the effects of Cm on the expression of sexual behavior, this extract at a high dose also improved the sexual performance of SExh males that recovered their ability to copulate.

The effects of* C. mexicana* shown here suggest that the constituents of this plant produce their stimulating effects by acting on central targets participating in the reversal of sexual exhaustion. A number of studies has demonstrated that the mesolimbic system comprises a fundamental target for installation of sexual satiation in males. The mesolimbic system is composed of mesencephalic structures, such as the ventral tegmental area (VTA), which contains dopaminergic neurons that send projections to limbic structures such as the nucleus accumbens (NAc). This system participates in providing rewards for natural (or artificial) stimuli, such as food and sexual activity [32]. It has been demonstrated that drugs facilitating dopaminergic transmission in the mesolimbic pathway reverse the sexual exhaustion [29]. These facts suggest that the constituents of Cm may engage in an interaction with dopaminergic system in the mesolimbic pathway. The comparison of the effects of Cm with those of yohimbine in the reversal of sexual satiation reinforces this idea. Yohimbine acts as an antagonist of *α*2-adrenergic receptors to increase noradrenaline release; this mechanism has been associated with the reversal of sexual exhaustion elicited by this drug [16]. In addition, it has been shown that the combination of subthreshold doses of apomorphine, a dopaminergic agonist, synergizes with subthreshold doses of yohimbine, to reverse sexual exhaustion. The simultaneous injection of haloperidol, a nonspecific dopamine receptor antagonist, with yohimbine, interferes with actions of the latter on sexual exhaustion [20]. Therefore, dopamine has been proposed as a crucial neurotransmitter in the action of yohimbine on sexual satiation [20]. Taking these data together, it is possible to suggest that the prosexual actions of Cm in SExh males are produced by changes in dopaminergic transmission in the mesolimbic system. This proposal is further supported by the results of sexual performance, although the actions of yohimbine on EL and NI were weaker than those elicited by Cm.

Stimulation of male sexual behavior by drugs could be associated with an increase in general locomotor activity. In the sexual behavior test, satiated males treated with the extracts of Cm and Td or with yohimbine exhibited a normal pattern of movement in the course of copulation, which was not different from that shown by males receiving the vehicle; that is, these rats did not demonstrate hyper- or hypoactivity nor stereotyped or circling movements. Results obtained in the OFT agree with these observations. Lower and medium doses of Cm were ineffective for restoring mating behavior, and only the lowest dose of Cm increased the ambulation of experimental animals. Additionally, the effective doses of Cm, Td, and yohimbine on sexual behavior test were not associated with stimulating motor effects. It can be suggested that there is no association between the actions of treatments on sexual activity and ambulatory activity.

It has been extensively documented that sexually satiated rats exhibit generalized hypersensitivity to drugs acting on different neurotransmitter systems [33]. The present data cannot preclude that doses of Cm higher than 320 mg/kg produce motor alterations; however, in addition to the quantitative evaluation, treatments and doses that stimulated sexual behavior did not produce undesirable effects in the OFT. Even more so, we do not have sufficient evidence to suggest that the hypersensitivity of sexually satiated males to drugs would include crude extracts. This question lies outside of the aim of our study, but it is possible to explain that, at least Td (the same employed herein) produces prosexual effects in sluggish or SExh male rats, but the former responded at lower doses (10 mg/kg) than sexually satiated males (80 mg/kg) on the same schedule [9, 10]. Also, it has been demonstrated that the standardized extracts used herein (Cm and Td) administered acutely (1,000–5,000 mg/kg) lacked effects on general locomotor activity, and only Cm at 5,000 mg/kg produced hyperactivity, kinking, and bristling tail in nonsexually experienced males [10, 21]. These elements were useful to determine that the effects of Cm and Td on sexual satiation are not affected by the motor alterations of rats resulting from sexual activity to satiation.

Our previous phytochemical investigations of the aerial parts of Cm led us to obtain a number of secondary metabolites; many of these are flavonoids in relatively high abundance [21, 22]. Flavonoids have many beneficial biological actions [9], among these prosexual effects [34], which could be associated with the synthesis and metabolism of sex hormones. Thus, daidzen, apigenin, and apigenin 7-glucoside (Ap-7-Glc) derivatives mimic the effects of estrogens [35], while flavones like chrysin [36], pinocembrin, and acacetin [37] produce a rise in the concentration of testosterone (T) in* in vivo* or* in vitro* models. In this regard, male sexual behavior is importantly stimulated by T in rats; in addition to T, its estrogenic metabolite, 17*β*-estradiol (E_2_), obtained through the aromatization of the androgen by the aromatase enzyme, is crucial for the expression of their sexual behavior [38]. The effects of sex hormones are produced by two mechanisms: steroids bind to intracellular receptors for the production of genomic, long-term effects or interact with the membrane receptor for the induction of short-term effects [39, 40]. Results obtained in the present study could agree with the idea of rapid effects evoked by sex steroids, because the prosexual response was obtained 60 min after the extracts were administered. However, more research is needed in order to know whether these extracts (or their components) are able to raise sex hormones in a shorter time period.

The most abundant compounds found in the aqueous extract of Cm are apigenin-7-*O*-*β*-D [(6′′-*O*-*p*-coumaroyl)]-glucoside, Ap-7-(6′′-coumaroyl-Glc), and three regioisomers in relative high abundance (93.4%). These latter are produced by an intermolecular rearrangement of 7-(6′′-coumaroyl-Glc). Flavonoid glucosides such as Ap-7-(6′′-coumaroyl-Glc) are susceptible to hydrolysis, leading first to the formation of apigenin-7-glucoside (Ap-7-Glc) and coumaric acid and then to free apigenin [41, 42]. It is interesting to note that these labile Ap-7-Glc derivatives are easily degraded to yield free apigenin during the isolation process, whereby these are not usually detected [10, 43]. In our previous study [21], we isolated, to our knowledge for first time, these Ap-7-(6′′-coumaroyl-Glc) from the aqueous extract of* C. mexicana*, while free apigenin was only detected in trace amounts. Free apigenin is present in several plants and is consistently reported to produce anxiolytic, antidepressant, and prosexual effects [41, 44]. However, species with anxiolytic activity, such as* Matricaria recutita* [45], lack prosexual effects. The above suggest that Ap-7-Glc derivatives contribute to the specific prosexual actions of Cm reported herein, rather than the free apigenin.

Additionally, in the Cm extract, the presence of some phenylpropanoids, such as caffeic and ferulic acids, was detected [21]. The former are proposed to modulate the nitric oxide (NO) pathway in some models of biological activity [46], while ferulic acid has been found to restore the content of brain monoamines in a model of monoamine-depletion in mice elicited by reserpine [47]. These neurotransmitters participate in sexual performance [9] and in the reversal of the sexual exhaustion state [16, 23, 29]. It is important to bear in mind that the phenomenon of sexual exhaustion and its reversal is very complex and is regulated by circuits beyond those participating in the expression of regular sexual behavior [17, 48]. In addition to monoamines and NO, other elements, such as opioids and excitatory aminoacids, take part in this phenomenon, which modulates neuronal excitability by different mechanisms downstream in cells [24, 29, 49]. Some of these pathways, such as the NO pathway, have been reported to participate in the prosexual effects of formulations from plants, such as the aqueous extract of* T. diffusa* [9].

In summary, data suggest the following: (1) the prosexual effects described here for Cm in behavior might be due to the activation of systems of rapid responses, such as neurotransmitters, that is, the catecholamines; (2) the prosexual effects of Cm are not associated with motor alterations; (3) reversal of the sexual decline is nearly always obtained with pure compounds [11]; thus, the positive results exposed here indicate an important stimulating effect of Cm on sexual behavior, and (4) the apigenin glycoside derivative, but not in its free form, could be responsible, at least in part, for the prosexual effect of Cm, as well as for that of Td. Specific studies are necessary to support these hypotheses.

In conclusion, the results presented herein demonstrate that Cm, as well as Td, reverses the inhibition of sexual behavior in SExh males. Differences in effective doses for improving the expression of sexual behavior and the properties of* C. mexicana* on sexual performance should be taken into account by users and traditional physicians, in order to produce the expected benefits.

## Figures and Tables

**Table 1:** Effect of the treatments on the number and percentage (%) of sexually exhausted male rats that expressed sexual behavior.

Treatments	Mounts	Intromissions	Ejaculation	Resuming
Vehicle, *n* = 20	9 (45)	7 (35)	7 (35)	0 (0)
Cm 80 mg/kg, *n* = 7	3 (43)	3 (43)	3 (43)	1 (14.3)
Cm 160 mg/kg, *n* = 10	6 (60)	6 (60)	6 (60)	1 (10)
Cm 320 mg/kg, *n* = 11	11 (100)	11 (100)	10 (90.9)	6 (54.5)
Td 80 mg/kg, *n* = 11	9 (81.8)	8 (72.7)	10 (90.9)	7 (63.6)
Yoh 2 mg/kg, *n* = 10	10 (100)	10 (100)	10 (100)	10 (100)

Left side of the table depicts the total number of subjects that were assigned to each group of treatment before the session of *ad libitum *copulation. Columns of the right side depict the number (and percentage) of sexually exhausted males that showed mounts, intromissions, and ejaculation or that resumed copulation in the test of sexual behavior. Treatments were administered 24 h after *ad libitum *copulation, and 1 h before the sexual behavior test. An aqueous extract of *C. mexicana* (Cm); an aqueous extract of *T. diffusa* (Td) and yohimbine (Yoh).

**Table 2:** Effect of the treatments on the number of mounts and intromission latency of sexually exhausted males rats that expressed sexual behavior.

Treatments	Number of mounts	Intromission latency (sec)
Veh	9.33 ± 2.35	60.75 ± 12.17
Cm 80 mg/kg	12.66 ± 6.06	91.33 ± 50.54
Cm 160 mg/kg	10.00 ± 3.89	63.83 ± 23.03
Cm 320 mg/kg	8.76 ± 3.44	239.76 ± 131.50
Td 80 mg/kg	16.10 ± 2.43	79.87 ± 17.48
Yoh 2 mg/kg	3.50 ± 0.60	45.70 ± 10.71

Data are expressed as mean ± S.E.M and the total number of subjects that were assigned to each group of treatment. An aqueous extract of *C. mexicana* (Cm); an aqueous extract of *T. diffusa* (Td) and yohimbine (Yoh).

**Table 3:** Effect of the treatments in male rats evaluated in the OFT.

Treatment	Number of counts/5 min
Vehicle	41.83 ± 3.05
Cm 80 mg/kg	60.50 ± 3.47
Cm 160 mg/kg	31.16 ± 5.35^*∗∗*^
Cm 320 mg/kg	44.00 ± 7.32
Td 80 mg/kg	34.16 ± 4.81^*∗∗*^
Yoh 2 mg/kg	45.16 ± 3.57

Rats were treated with aqueous extracts of *C. mexicana* (Cm), *T. diffusa* (Td), or yohimbine (Yoh). Data are expressed as mean ± S.E.M. Paired comparisons in groups treated with Cm were done with the correction of Bonferroni. Other comparisons were done with the Mann Whitney *U* test. ^*∗∗*^ *P* < 0.01 versus Cm at 80 mg/kg.
